# Hemodynamic monitoring: basic principles in operation room and intensive care unit

**DOI:** 10.1007/s10877-025-01397-6

**Published:** 2026-01-06

**Authors:** Martin Mirus, Bernd Saugel, Peter M. Spieth

**Affiliations:** 1 Department of Anesthesiology and Intensive Care Medicine, University Hospital Dresden, Technische Universität Dresden, Fetscherstr. 74, 01307 Dresden, Germany; 2 Department of Anesthesiology, Center of Anesthesiology and Intensive Care Medicine, University Medical Center Hamburg-Eppendorf, Hamburg, Germany

**Keywords:** Anesthesia, Hemodynamic, Cardiac output, Goal directed therapy, Intensive care medicine, Oxygen delivery

## Abstract

To synthesize physiological principles and practical monitoring strategies for perioperative and intensive care hemodynamics, addressing two questions: which variables should be monitored, and what targets should be pursued to optimize outcomes. The review is educational in scope and highlights instrumentation details and clinical applications.Narrative, physiology-anchored review of oxygen delivery, venous return (VR), and regulation of mean arterial pressure (MAP) as a derived target. Reviews for educational purposes are included, with emphasis on instrumentation principles and clinical use cases. Invasive and non-invasive modalities are compared. Evidence from goal-directed therapy (GDT) trials in operating room and intensive care contexts is summarized to link physiology with therapy.(1) adequacy of oxygen delivery cannot be judged from MAP alone; MAP reflects the interaction of cardiac output (CO), systemic vascular resistance (SVR), and right atrial pressure. (2) VR depends on effective circulating volume, venous compliance, and mean systemic filling pressure. (3) Microcirculatory assessment remains limited; macrocirculatory surrogates and biomarkers provide guidance but have constraints. (4) Device outputs labeled identically are not interchangeable; calibration strategy and physiological assumptions are decisive. (5) GDT improves processes and may benefit selected high-risk patients, but large trials show mixed effects on mortality.Effective hemodynamic management requires physiology-based reasoning: identify the limiting factor, then select monitoring tools and therapeutic targets accordingly. MAP must be interpreted with CO, SVR, and RAP. Individualized, dynamic targets and trend-based responses outperform fixed thresholds. Embedding ultrasound skills, fluid-responsiveness testing, and calibrated device interpretation are levers to translate monitoring into safer care.

## Key Messages

1. Physiological understanding is fundamental: Effective hemodynamic management requires a robust grasp of cardiovascular physiology, particularly the interplay between preload, afterload, contractility, and venous return.
2. MAP is not a standalone target: MAP reflects the interaction between flow and resistance and should not be interpreted in isolation from CO or SVR. 
3. Individualized hemodynamic targets are essential: Standard reference ranges may not apply to all clinical scenarios; patient-specific factors and contextual variables must guide goal-directed therapy.
4. Monitoring technology is a tool – not a solution: Whether calibrated, auto-calibrated or uncalibrated, invasive or non-invasive, monitoring modalities must be selected and interpreted based on the clinical question, not merely on technological availability.

## Introduction

The advent of general anesthesia in the 19th century not only revolutionized surgical practice but also underscored the necessity of intraoperative monitoring to prevent anesthesia-related complications. While early discussions focused on the necessity of monitoring itself [1, 2], contemporary debates have shifted toward identifying the most appropriate monitoring modalities, relevant physiological variables, and evidence-based target values [3–6].

Hemodynamic monitoring - although only one component of perioperative care - has become an established cornerstone of patient management in both the operating room (OR) and intensive care unit (ICU). Timely recognition and correction of circulatory disturbances are essential to prevent organ dysfunction and improve clinical outcomes. Despite advances in monitoring technology, two fundamental questions remain as relevant today as they were more than a century ago: Which physiological variables should guide clinical management, and what target thresholds should be aimed for to optimize outcomes?

### Which variables should we monitor?

The number of measurable hemodynamic variables continues to grow, driven by advances in both physiological understanding and monitoring technology. However, the assumption that deeper physiological insight automatically leads to meaningful monitoring is flawed. For instance, although it is well recognized that microcirculatory disturbances may persist in septic shock despite preserved macrocirculatory variables, standardized tools for routine clinical assessment of the microcirculation are still lacking [4, 7–9]. Conversely, a variety of devices offer variables such as cardiac power output or contractility indices, the physiological and clinical relevance of which remain poorly validated [10, 11]. It must also be taken into account that, even if manufacturers label measured variables identically, this does not imply that the values obtained from different devices are interchangeable [12–15]. Answering the question of which variables to monitor requires a foundational understanding of the physiological processes under assessment.

### What target values should we aim for?

Objective monitoring allows for the verification of whether patients attain predefined physiological targets. For instance, a cardiac index (*CI*) between 2.5 and 3.5 L/min/m² is generally considered to lie within the ‘normal range’. However, such normative values do not necessarily represent optimal therapeutic targets across all clinical scenarios. An 82-year-old woman undergoing posterior spinal fusion for an osteoporotic fracture may not benefit from the same hemodynamic targets as a 62-year-old man undergoing hemicolectomy for ischemic colitis in the context of pneumococcal septic shock. In 1988, Shoemaker et al. [16] demonstrated that targeting supranormal values of *CI*, oxygen delivery (*DO₂*), and oxygen consumption (*VO₂*) improved survival in high-risk surgical patients, thereby challenging the adequacy of standard values in critically ill populations. Recent studies have not been able to confirm this previously reported clear benefit of hemodynamic parameter optimization [17–19]. In contrast to the perioperative studies by Shoemaker et al. [16], subsequent randomized trials in mixed ICU populations tested whether maximizing *DO₂* or achieving supranormal *CI* improves outcomes. Hayes et al. [20] targeted supranormal *DO₂* using dobutamine in critically ill patients but did not observe a mortality benefit and reported increased complications in some subgroups. Gattinoni et al. [21] likewise compared strategies aimed at supranormal *CI* or *SvO₂* against conventional targets and found no reduction in morbidity or mortality. These data suggest that indiscriminate pursuit of supranormal *DO₂* in heterogeneous ICU cohorts may be ineffective or even harmful. Importantly, the perioperative setting differs from the ICU. In the operating room, the hemodynamic insult is usually time-limited and predictable, with a clearly defined high-risk window around major surgery. In the ICU, patients often have sustained, complex circulatory derangements driven by sepsis, cardiogenic shock, or respiratory failure, with prominent microcirculatory and mitochondrial abnormalities. Consequently, aggressive *DO₂* maximization that may be defensible in selected high-risk surgical patients cannot simply be extrapolated to unselected critically ill ICU populations. Taken together, these studies underline that optimal hemodynamic targets are context-dependent: strategies that may be beneficial in carefully selected high-risk surgical patients can fail or even cause harm when generalized to heterogeneous ICU populations.

### The challenge

A central challenge in anesthesiology and critical care still lies in identifying clinically meaningful variables and determining appropriate target ranges for each individual patient [22]. These decisions cannot be standardized but must integrate patient-specific factors (e.g., age, comorbidities, baseline function) and clinical context (e.g., emergency surgery vs. sepsis) [4, 23]. Meeting this challenge requires more than technical competence; it demands a thorough understanding of cardiovascular physiology and the contextual interpretation of hemodynamic data. This article aims to support that understanding by providing a concise overview of core physiological principles and their practical application in monitoring-guided clinical decision-making.

## Key physiological mechanisms underlying hemodynamic monitoring

### Oxygen delivery and consumption

**Fig. 1:**
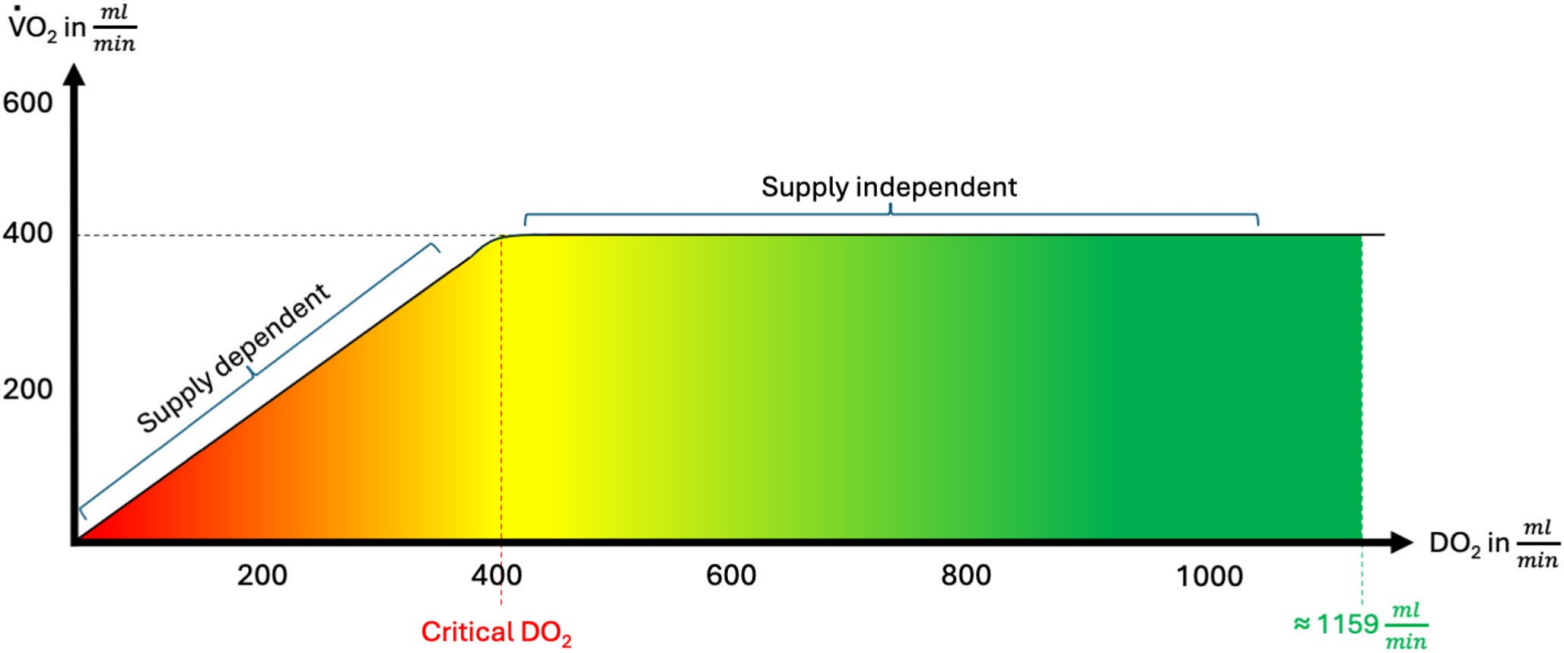
Relationship Between *DO₂* and *VO₂*

Sustained organ function relies on adequate oxygen metabolism. At the cellular level, this depends on sufficient oxygen delivery, preserved mitochondrial function, and effective clearance of metabolic by-products [24]. As direct assessment of microcirculatory function remains technically challenging [24–27], macrocirculatory variables are used as surrogate indicators to estimate whether *DO₂* meets the metabolic demand in a given clinical context (Fig. 1). This representation constitutes a simplification, as oxygen demand may continue to rise in critically ill patients, and various other factors can influence the *VO₂/DO₂* relationship [28, 29]. Nonetheless, the illustration emphasizes the importance of considering a critical threshold of *DO₂*, which is inherently individual and context-dependent. Global oxygen delivery is only one side of the equation. Whether cellular oxygen demand is met depends also on peripheral extraction, commonly expressed as the oxygen extraction ratio (O₂ER = *VO₂/DO₂)* and reflected by the arterio-venous oxygen content difference. In states of pure supply dependency, impaired *DO₂* leads to increased extraction and widening of the arterio-venous O₂ difference until a critical *DO₂* is reached. Beyond this point, further falls in *DO₂* cause a decline in *VO₂* and manifest tissue hypoxia. In sepsis and other inflammatory conditions, mitochondrial dysfunction and microcirculatory shunting may blunt peripheral extraction (‘cytopathic hypoxia’), leading to paradoxically high *ScvO₂* values despite inadequate tissue oxygenation. In such scenarios, lactate and the arterial–venous *CO₂* difference (*avDCO₂*) become particularly important complementary markers [30]. Contemporary hemodynamic theory rests on two fundamental physiological assumptions: (1) cardiac output (*CO*) is the primary determinant of tissue perfusion, and (2) blood is the principal carrier of oxygen. Consequently, variables affecting both blood flow and arterial oxygen content must be monitored. However, the adequacy of oxygen delivery alone does not confirm that cellular oxygen demand is met nor does a normal *ScvO*_*2*_ necessarily rule out impaired peripheral extraction. Clinical indicators of an *VO₂/DO₂* - mismatch can include elevated lactate concentrations, reduced central venous oxygen saturation (*ScvO₂*), and an increased arterial–venous carbon dioxide difference (*avDCO₂*), although these variables exhibit limitations [24, 31]. When oxygen delivery is insufficient to meet metabolic requirements – either globally or regionally – shock ensues.

This figure illustrates the biphasic relationship between *DO₂* and *VO₂*. In the supply-dependent region, *VO₂* increases proportionally with *DO₂*, indicating that tissue oxygen consumption is limited by delivery. Once the critical *DO₂* threshold is reached (~ 400 ml/min), *VO₂* plateaus and becomes supply-independent, reflecting maximal oxygen extraction capacity under normal physiological conditions. The transition to supply independence indicates adequate oxygen availability, beyond which increases in *DO₂* no longer enhance *VO₂*. The upper boundary (~ 1159 ml/min) marks the physiological average of *DO₂*. This curve is essential in understanding tissue oxygenation and guiding hemodynamic optimization in critically ill patients.

*DO₂*: Oxygen delivery; *VO₂*: Oxygen consumption.

### Determinants of *CO*

**Fig. 2:**
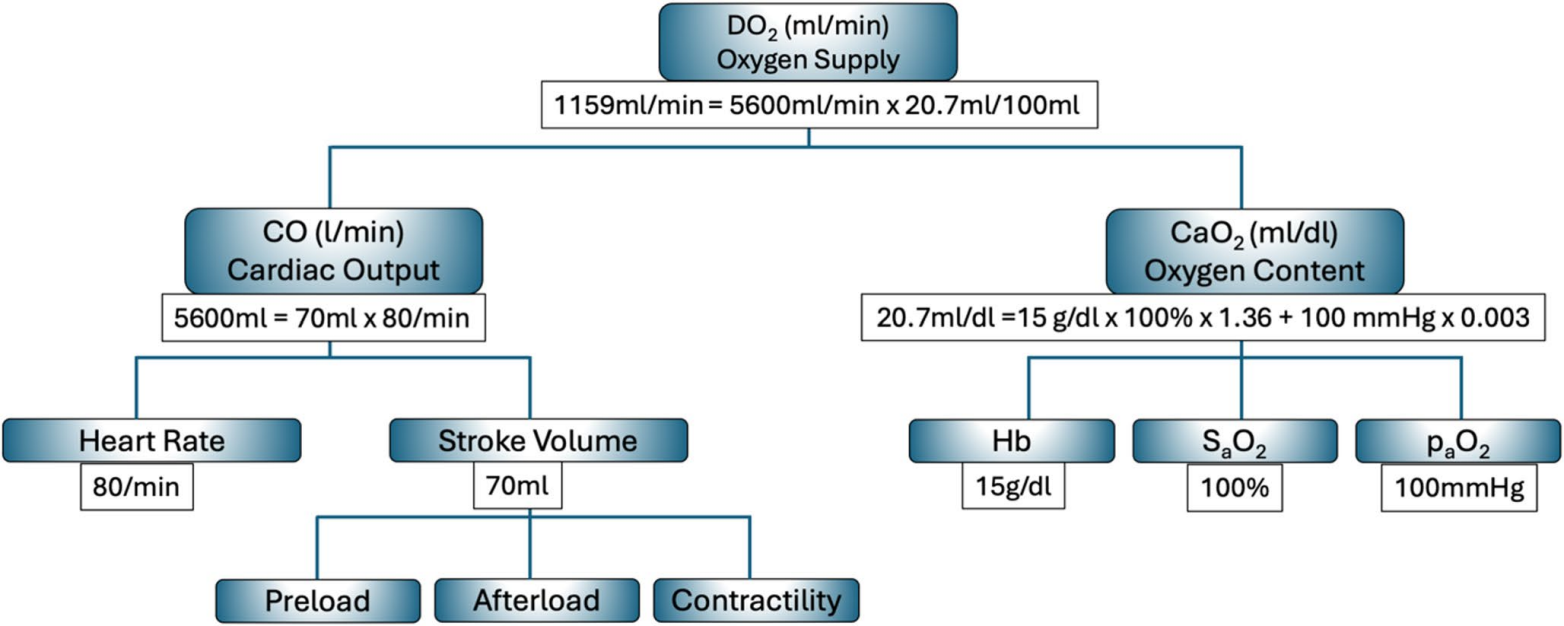
Determinants of *DO₂*: Components and Relationships

**Fig. 3:**
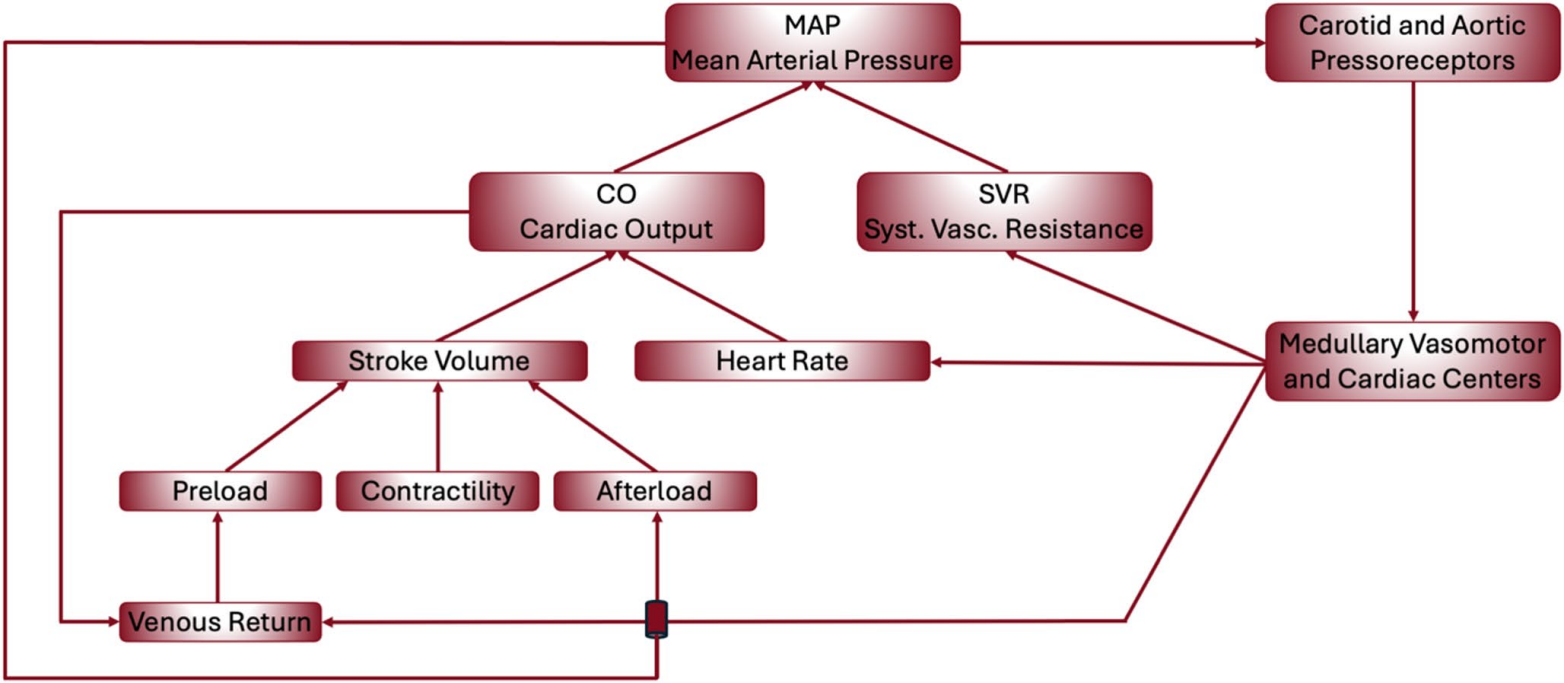
Regulation of *MAP* via *CO* and *SVR*

The core determinants of hemodynamic physiology include *CO*, preload, myocardial contractility, and afterload (Fig. 2). Preload refers to ventricular end-diastolic volume, is governed by venous return, and influences stroke volume via the Frank-Starling mechanism – within physiological limits, greater preload results in increased stroke volume. Afterload represents the load faced by the ventricle during ejection. From a physiological perspective, left ventricular afterload is best described by aortic input impedance, which integrates steady resistance, large-artery compliance, and wave reflections. Clinically, systolic arterial pressure and calculated SVR are commonly used surrogate markers of afterload. SVR, however, is a lumped steady-state parameter reflecting arteriolar resistance and vasomotor tone rather than the full pulsatile load imposed on the left ventricle. Contractility denotes the intrinsic contractile strength of the myocardium, independent of preload and afterload. Importantly, *DO₂* depends not only on *CO* but also on hemoglobin concentration and arterial oxygen saturation. Although mean arterial pressure (*MAP)* is commonly used as a surrogate marker for tissue perfusion, it is important to recognize that *MAP* is influenced by multiple physiological parameters and does not directly reflect tissue perfusion (Fig. 3).

This schematic illustrates the physiological components contributing to *DO₂*, defined as the product of *CO* and *CaO₂*. *CO* is determined by heart rate and stroke volume, with stroke volume influenced by preload, afterload, and myocardial contractility. *CaO*₂ depends on hemoglobin concentration (Hb), arterial oxygen saturation (SaO₂), and the partial pressure of oxygen in arterial blood (PaO₂), as expressed by the formula:

$$CaO{\text{ }} = {\text{ }}\left( {Hb{\text{ }} \times {\text{ }}1.36{\text{ }} \times {\text{ }}SaO} \right){\text{ }} + {\text{ }}\left( {0.003{\text{ }} \times {\text{ }}PaO} \right).$$

The diagram quantifies a typical example yielding a *DO₂* of approximately 1159 ml/min, based on a cardiac output of 5600 ml/min and a *CaO₂* of 20.7 ml/dl. This figure highlights the key variables that must be considered when assessing or optimizing systemic oxygen delivery in clinical practice.

*CO*: Cardiac Output; *CaO₂*: arterial oxygen content; *DO₂*: Oxygen delivery; *VO₂*: Oxygen consumption; Hb: hemoglobin concentration; SaO₂: arterial oxygen saturation.

This diagram illustrates the physiological determinants and regulatory pathways involved in maintaining *MAP*. *MAP* is determined by *CO* and *SVR*. *CO* is influenced by stroke volume and heart rate, with stroke volume further modulated by preload, contractility, and afterload. *VR* contributes to preload and is influenced by vascular tone and intravascular volume. *MAP* is sensed by carotid and aortic baroreceptors, which transmit signals to medullary vasomotor and cardiac centers to adjust autonomic output and maintain homeostasis. This integrated feedback loop ensures short-term blood pressure regulation and effective perfusion of vital organs. Adapted from [32].

*CO*: Cardiac Output; *MAP*: Mean arterial pressure; *SVR*: systemic vascular resistance.

### Electrical circuit analogy

The analogy to an electrical circuit is frequently used to conceptualize hemodynamic physiology, although it oversimplifies several important aspects [33]. In an electrical system, three fundamental variables are considered: voltage (*U*), defined as the potential difference between two points; current (*I*), representing the flow of electric charge per unit time; and resistance (*R*), describing the voltage required to sustain a current of one ampere (Table 1). Applied to the circulatory system, this analogy yields a functional model in which mean perfusion pressure (*MPP*) - defined as *MAP* minus right atrial pressure (*RAP*) and corresponds to the driving pressure, or “voltage”, for systemic perfusion; *CO* represents the “current”, or flow of blood; and *SVR* reflects the opposition to flow, analogous to electrical “resistance”. This relationship is mathematically represented by a modified form of Ohm’s law:

**Table 1:** Analogy between electrical and hemodynamic systems

Electrical System	Hemodynamic System	Concept Explanation	
Voltage (V)	Mean Perfusion Pressure (MPP = MAP – RAP)		Driving force for flow
Current (I)	Cardiac Output (CO)		Volume of flow per unit time
Resistance (R)	Systemic Vascular Resistance (SVR)		Opposition to flow
Ohm’s Law: V = I × R	Hemodynamic Law: MAP = CO × SVR		Flow determined by pressure gradient and resistance
Battery (energy source)	Heart (pressure generator)		Provides the energy to drive flow
Electrical circuit	Circulatory system		Closed loop system
No current without voltage	No flow without perfusion pressure		Flow only occurs with a pressure gradient
Resistance impedes current	Vascular resistance limits blood flow		Vasoconstriction ↑ SVR; vasodilation ↓ SVR

This table illustrates the analogy between electrical and hemodynamic systems to clarify fundamental physiological principles of blood flow regulation. Key parameters such as voltage, current, and resistance are mapped to their hemodynamic counterparts: mean perfusion pressure (MAP – RAP), cardiac output (CO), and systemic vascular resistance (SVR), respectively. Just as in an electrical circuit, blood flow in the circulatory system is driven by a pressure gradient and limited by resistance, following a hemodynamic equivalent of Ohm’s law. MPP: Mean Perfusion Pressure; MAP: Mean Arterial Pressure; RAP: Right Atrial Pressure (Central Venous Pressure); CO: Cardiac Output; SVR: Systemic Vascular Resistance

1 $$\:MAP\:-RAP\:=CO\:\times\:\:SVR$$

This formulation emphasizes that arterial pressure is not an independent physiological target, but rather a consequence of the interaction between flow and resistance. Consequently, changes in *MAP* and *RAP* may reflect alterations in either *CO*, vascular tone, or both [34, 35]. Accordingly, *MAP* should not be used as a surrogate for *CO* [4, 35]. A clear understanding of this relationship is essential for goal-directed hemodynamic management, as it enables clinicians to distinguish between hypotension due to impaired pump function (e.g., reduced *CO*) and hypotension due to distributive causes (e.g., decreased *SVR*), thereby guiding appropriate therapeutic interventions. For most organs, *MAP* is an appropriate approximation of perfusion pressure. An important exception is left ventricular subendocardial perfusion, which occurs mainly during diastole and is determined by the gradient between diastolic aortic pressure and left ventricular end-diastolic pressure. Thus, low diastolic pressure and elevated LV filling pressures may jeopardize coronary perfusion even when MAP appears acceptable.

### Beyond ohm’s law: limitation of electrical circuit analogy

The simplified analogy to Ohm’s law, while useful for didactic purposes, fails to capture the full complexity of circulatory physiology. It omits key determinants such as intravascular volume and vascular compliance [33] – factors that critically influence both *CO* and blood pressure. Furthermore, it suggests that the heart functions as an autonomous pressure generator, disregarding the fact that *CO* is ultimately limited by venous return (*VR*) [36].

### Guyton’s model: integrating *VR* and *CO*

A more physiologically accurate framework is provided by the Guyton model of circulatory equilibrium, which emphasizes that *CO* and *VR* must be equal at steady state: $\:VR=CO$. *VR* is determined by the pressure gradient between the upstream ‘reservoir’ pressure of the systemic vasculature and the right atrial pressure (*RAP*), divided by the resistance to venous return ($\:{R}_{v}$):

2 $$\:VR=CO=\:\frac{MSFP-RAP}{{R}_{v}}=HR\times\:SV$$

Here, mean systemic filling pressure (*MSFP)* represents the pressure within the systemic vasculature that would be measured if the heart were stopped and flow ceased. So, the pressure in the systemic circulation ignoring the heart and pulmonary circulation, in the absence of flow [37, 38]. *MSFP* is a function of effective circulating volume and venous tone (i.e., venous compliance). $\:{R}_{v}$ reflects the aggregate resistance along the venous pathway back to the right atrium. Mean circulatory filling pressure (*MCFP*) extends this concept to the entire closed circulation, including heart and lungs, under no-flow conditions. Although *MSFP* and *MCFP* are closely related, they are not identical constructs. In his experiments with dogs, Guyton found that the normal *MCFP* was around 6.3 ± 0.94 mmHg [39]. This is the assumed *MCFP* for humans as well. An increase in blood volume or venoconstriction (decreased compliance) raises *MSFP*, thereby promoting *VR* and, ultimately, *CO*. By substituting this relationship into the *MAP* equation, we obtain a more comprehensive expression:

3 $$\:MAP=\left(\:\frac{MSFP-RAP}{{R}_{v}}\:\times\:\:SVR\right)+RAP$$

This equation illustrates that *MAP* is not only a function of cardiac and vascular dynamics, but also of the interaction between preload determinants and *RAP*.

### Determinants of *MSFP*

This is connected to the foundational concept of distinction between stressed and unstressed volume [40]. The stressed volume refers to the portion of circulating blood that stretches the vascular wall and thus generates pressure. This volume remains relatively constant over short periods but can be altered by fluid shifts. The pressure within the vascular system results from the distension of its elastic walls by this volume. Importantly, pressure results from volume – not the other way around. Pressure may exist even in the absence of flow; however, flow only occurs when a pressure gradient exists between two vascular compartments, with blood moving from a region of higher to lower pressure [41]. The *MSFP* is, in itself, dependent on two major factors:

4 $$\:MSFP\sim\frac{{V}_{eff}}{{C}_{v}}$$

where $\:{V}_{eff}$ is the effective circulating volume and $\:{C}_{v\:}$is the compliance of the venous system. Thus, an increase in venous tone (i.e., reduced compliance via sympathetic stimulation) or a fluid bolus will elevate $\:MSFP$ enhance *VR*, and potentially increase *MAP* – provided that the heart is able to accommodate the increased preload. Clinically, *VR* can be modulated either by changing the pressure gradient (*MSFP – RAP*) or by modifying $\:{R}_{v}$. Fluid boluses and venoconstrictors (e.g. norepinephrine) increase *MSFP* and thus the driving pressure for *VR*, provided *RAP* does not rise to the same extent. Venodilators and neuraxial blockade decrease venous tone, reducing *MSFP* and *VR*. Mechanical ventilation and high PEEP elevate intrathoracic pressure, which can increase *RAP* and thereby decrease the gradient, particularly in hypovolemic patients. Increases in intra-abdominal pressure or severe right ventricular failure effectively raise $\:{R}_{v}$ by adding resistance to venous return. Understanding which component is limiting venous return is critical to choosing between fluids, vasopressors, inodilators, or ventilator adjustments.

### Implications for clinical practice

Understanding this interplay is critical in the ICU and operating room. For example:

- In septic shock, decreased *SVR* and altered venous tone reduce both *MAP* and *MSFP*, necessitating the use of vasopressors to restore vascular tone and volume resuscitation to support preload.
- In hypovolemia, low *V*_*eff*_ results in reduced *MSFP*, thereby limiting *VR* and *CO* regardless of cardiac function.

Consequently, interventions aimed at increasing *MAP* must be selected based on the specific limiting factor in a given patient: volume, tone, contractility, or afterload.

## Clinical and paraclinical assessment of hemodynamics

Despite the growing availability of advanced monitoring technologies, clinical assessment remains central to hemodynamic evaluation. Key bedside indicators of tissue hypoperfusion include altered mental status, dyspnea, angina, cold extremities, prolonged capillary refill time (*CRT*), and decreased urine output. These signs reflect consequences of already impaired perfusion, not its etiology. Among these, *CRT* has gained particular attention as a simple, rapid, and reproducible indicator of peripheral perfusion and a potential bridge between macrocirculation and microcirculation. In septic shock, abnormal *CRT* has been associated with microcirculatory disturbances, loss of hemodynamic coherence, and worse outcomes, while *CRT*-guided resuscitation strategies have shown promise in pilot and randomized studies. A normalized *CRT* despite low or borderline *MAP* may indicate preserved peripheral perfusion and argue against further escalation of vasopressors, whereas a persistently prolonged *CRT* despite ‘normal’ macrocirculatory indices can unmask residual microcirculatory hypoperfusion and prompt reassessment of the therapeutic strategy. Evidence from the trials ANDROMEDA-SHOCK [42] and the recent ANDROMEDA-SHOCK-2 [43] strengthens the concept that peripheral perfusion is a physiologically meaningful resuscitation target. In these studies, resuscitation strategies guided primarily by *CRT* were associated with faster resolution of metabolic abnormalities, fewer vasopressor requirements, and signals of improved organ function. ANDROMEDA-SHOCK-2 [43] further suggests that *CRT*-targeted resuscitation reduces duration of organ support by identifying patients in whom microcirculatory adequacy has already recovered despite persisting abnormalities in global variables such as *MAP* or lactate. A normalized CRT despite low or borderline MAP may thus indicate preserved peripheral perfusion and argue against further escalation of vasopressors, whereas a persistently prolonged CRT despite “normal” macrocirculatory indices can unmask residual microcirculatory hypoperfusion and prompt a reassessment of the therapeutic strategy.

CRT should be assessed in a standardized fashion (e.g. 5-second compression of the distal phalanx at heart level with a threshold of > 3 s considered abnormal in adults). Lactate serves as a sensitive marker of hypoperfusion but is influenced by confounders and must be interpreted in light of factors influencing production and clearance (Table 2) [44, 45]. Besides hepatic dysfunction, β-adrenergic stimulation, seizures, and regional ischemia, renal replacement therapy (RRT) can decrease circulating lactate through extracorporeal clearance [46]. A declining lactate concentration in a patient on RRT therefore does not automatically reflect improved tissue perfusion and lacks real-time responsiveness. Although intrinsic lactate overproduction cannot be masked, lactate declines during renal replacement therapy may represent extracorporeal clearance rather than a genuine improvement in tissue perfusion [47]. Therefore, clinical signs must be complemented by objective hemodynamic data to identify shock states and tailor therapy.

**Table 2:** Factors influencing lactate levels beyond tissue hypoxia

Category	Examples/Mechanisms
Increased production	
Adrenergic stimulation	Sepsis, Pain, Trauma, Catecholamines → β2-mediated aerobic glycolysis ↑
Increased metabolic demand	Seizures, shivering, strenuous exercise
Malignancies	Warburg effect (aerobic glycolysis in tumors)
Toxin-induced	Ethanol, methanol, cyanide, salicylates → interfere with mitochondrial respiration
Decreased clearance	
Liver Failure	Primary site of lactate metabolism impaired → reduced lactate clearance → accumulation despite normal perfusion
Shock states	Liver hypoperfusion limits clearance
Other causes	
Thiamine deficiency	Blocks pyruvate dehydrogenase → favors lactate formation
Mitochondrial disorders	Congenital or acquired dysfunction in oxidative phosphorylation
Alkalosis	Alters lactate-pyruvate equilibrium; can reduce clearance
Drugs	Metformin (especially in renal failure), SGLT2i, linezolid, nucleoside analogues, antiretrovirals
RRT	Extracorporeal clearance of lactate can lower measured levels independent of macrocirculatory or microcirculatory improvement

This table summarizes key categories and mechanisms contributing to elevated lactate levels in clinical settings. Lactate accumulation can result from increased production (e.g., due to adrenergic stimulation or metabolic demand), impaired clearance (e.g., liver dysfunction or hypoperfusion), or other causes such as mitochondrial dysfunction or medication effects. β2: Beta-2 adrenergic receptor; SGLT2i: Sodium-glucose cotransporter 2 inhibitors; RRT: Renal replacement therapy

### Invasive and Non-Invasive monitoring techniques

A range of tools is available for hemodynamic assessment, broadly categorized into invasive and non-invasive modalities. Both methods further be divided into calibrated (i.e. external calibrated), auto-calibrated (i.e. internal calibrated), and uncalibrated tools [48–51]. Invasive methods typically require invasive arterial pressure monitoring, as hemodynamic variables are derived from the analysis of the arterial pressure waveform using manufacturer-specific pulse wave or pulse contour algorithms.

Auto-calibrated techniques estimate these variables solely through built-in algorithms, which – broadly speaking – rely on comparisons with reference populations embedded in the system [48]. In contrast, calibrated techniques do not use such population-based approximations. Instead, they determine hemodynamic variables for the individual patient through calibration using a one-time administration of an indicator substance. The kinetics of this indicator – measured in the arterial system – are used to calculate patient-specific hemodynamic variables. This process requires a venous access route for indicator injection; in most systems, administration near the right atrium is necessary, necessitating the placement of a central venous catheter (CVC). To detect the indicator in the arterial system, an arterial catheter is also required. While the earliest descriptions of this indicator technique involved dye dilution, today the most commonly used indicator is a transient change in blood temperature: *thermodilution* techniques. Accordingly, temperature changes are detected by a thermistor integrated into the arterial catheter. Based on the thermistor’s location – either in the pulmonary arterial or systemic arterial circulation – two techniques are distinguished: pulmonary artery and transpulmonary thermodilution. In many cases, the temperature sensor is integrated into an invasive arterial pressure catheter, allowing both pressure and temperature measurement via a single access point. The measured pressure corresponds either to the pulmonary arterial pressure or to the systemic arterial pressure, depending on catheter placement. To ensure that hemodynamic variables are available beyond the brief window during which the indicator is administered, calibrated systems typically, but not mandatory, also provide continuous measurement of hemodynamic variables independently of indicator kinetics. This is achieved through pulse wave or pulse contour analysis, similar to uncalibrated methods. However, in calibrated systems, these analyses are adjusted based on the most recent indicator-derived calibration, thereby improving individual accuracy. To maintain this accuracy, regular recalibration using repeated indicator measurements is required. Uncalibrated systems offer a minimally invasive approach to derive *CO* and other hemodynamic variables from the arterial pressure waveform without any biometric data [49, 52, 53]. Table 3 summarizes essential hemodynamic variables, their methods of estimation, and corresponding physiological values.

**Table 3:** Comprehensive overview of key hemodynamic variables in thermodilution monitoring

Parameter	Unit	Assessment/Calculation	Physiological Role	Physiological Range
Cardiac Output (CO)	L/min	HR x SV	Global blood flow from the heart	4–8
Cardiac Index (CI)	L/min/m²	CO/BSA	CO indexed to body surface area for individual assessment	2,5 − 3,5
Oxygen Content (CaO_2_)	ml/dl	(1.36×Hb×SaO_2_)+(0.0031×PaO_2_ in mmHg)	Represents oxygen carried in arterial blood	21
Oxygen Supply (DO_2_)	ml/min	COx[(1.36×Hb×SaO_2_)+(0.0031×PaO_2_ in mmHg)]	Total oxygen delivery to the tissues per minute	950–1150
ScvO_2_	%	Direct measurement (PAC)	Surrogate for systemic oxygen balance (O_2_ delivery vs. demand)	70–80%
Systemic Vascular Resistance (SVR)	dyn·s·cm⁻⁵	80 × (MAP − CVP)/CO	Afterload of left ventricle; reflects vascular tone	800–1200
Systemic Vascular Resistance Index (SVRI)	dyn·s·cm⁻⁵·m^2^	80 × (MAP − CVP)/CI	SVR adjusted for body surface area	1400–2500
Pulmonary Vascular Resistance (PVR)	dyn·s·cm⁻⁵	80 × (MPAP − PAOP)/CO	Afterload of right ventricle; pulmonary vascular tone	20–130
Pulmonary Vascular Resistance Index (PVRI)	dyn·s·cm⁻⁵·m^2^	80 × (MPAP − PAOP)/CI	PVR adjusted for body surface area	255–285
Central Venous Pressure (CVP)	mmHg	Direct measurement (CVC)	Right atrial pressure; reflects preload and volume status	2–8
Pulmonary Artery Pressure (PAP)	mmHg	Direct measurement (PAC)	Pressure in pulmonary artery; reflects RV function and resistance	15–25/8–15
Mean Pulmonary Artery Pressure	mmHg	Direct measurement (PAC)	Average pressure in pulmonary artery; used in PH diagnosis	10–20
Pulmonary Artery Occlusion Pressure (PAOP)	mmHg	Measured during balloon inflation	Reflects left atrial pressure; surrogate for LV preload	6–12
Global End-Diastolic Volume (GEDV)	mL	ITTV − PTV = CO × (MTt − DSt)	Volumetric preload indicator	800–1200
Global End-Diastolic Volume (GEDVI)	mL/m^2^	(ITTV - PTV)/BSA	GEDV indexed to body surface area	680–800
Intrathoracic Blood Volume Index (ITBVI)	ml/m^2^	GEDV × 1.25	Indicator of central blood volume	850–1000
Extravascular Lung Water (ELW)	mL	ITTV − ITBV	Marker of pulmonary edema	< 500 ml
Extravascular Lung Water Index (ELWI)	mL/kg	(ITTV - ITBV)/bodyweight	Marker of pulmonary edema	< 10 ml/kg
Stroke Volume Index	ml/m^2^	SV/BSA	Output per heart beat	40–60
Stroke Volume Variation (SVV)	%	(SVmax − SVmin)/SVmean × 100	Dynamic preload indicator, fluid responsiveness predictor	≤ 10–12
Pulse Pressure Variation (PPV)	%	(PPmax − PPmin)/PPmean × 100	Dynamic preload indicator; assesses fluid responsiveness	≤ 13%
Right Ventricular Ejection Fraction (RVEF)	%	SV_RV/EDV_RV (via PAC)	Assesses right ventricular systolic function	≥ 45%

This table provides a detailed summary of essential hemodynamic parameters used in thermodilution and advanced hemodynamic monitoring. It includes units of measurement, calculation methods, physiological significance, and normal reference ranges. The listed parameters support clinical interpretation in critically ill patients and perioperative settings by describing preload, afterload, contractility, and global perfusion. CO: Cardiac Output; CI: Cardiac Index; Hb: Hemoglobin; SaO₂: Arterial oxygen saturation; PaO₂: Arterial oxygen partial pressure; MAP: Mean Arterial Pressure; CVP: Central Venous Pressure; MPAP: Mean Pulmonary Artery Pressure; PAOP: Pulmonary Artery Occlusion Pressure; ITTV: Intrathoracic Thermal Volume; GEDV: Global End-Diastolic Volume; ITBV: Intrathoracic Blood Volume; EVLW: Extravascular Lung Water; PPV: Pulse Pressure Variation; RVEF: Right Ventricular Ejection Fraction; ITTV: Intrathoracic Thermal Volume = the total thermal volume within the thorax; PTV: Pulmonary Thermal Volume = the thermal volume of the pulmonary circulation; MTt: Mean Transit Time = average time of thermal indicator transit; DSt: Downslope Time = exponential decay time of the thermodilution curve

### Invasively calibrated thermodilution: pulmonary artery vs. transpulmonary methods

Pulmonary artery thermodilution involves measurement of temperature changes within the pulmonary circulation using a pulmonary artery catheter (PAC). The cold indicator is injected into the right atrium and passes through the right ventricle into the pulmonary artery, where the thermistor detects the resulting temperature change – typically in the pulmonary trunk or one of its branches, depending on catheter position. Formally, the PAC measures the *CO* generated by the right ventricle, as the left ventricle does not contribute directly to the thermodilution curve. Therefore, a reduction in *CO* caused by isolated left ventricular (LV) failure may remain undetected. In such cases, LV dysfunction can only be inferred from secondary variables, such as elevated pulmonary artery wedge pressure (*PAWP*) or increased pulmonary artery pressures (*PAP*). The PAC additionally enables direct measurement of *PAP*, *PAWP*, and mixed venous oxygen saturation (*SvO₂*). Contemporary PA catheters incorporate advanced sensors and signal processing that enable continuous *CO* monitoring and right ventricular performance metrics. In addition to thermal filament-based continuous thermodilution [54], experimental and early clinical systems have used right ventricular pressure waveforms and mathematical modelling to derive *CO* in near real time [55]. These developments illustrate how invasive pressure monitoring and waveform analysis are increasingly integrated to provide continuous, high-fidelity hemodynamic information.

Transpulmonary thermodilution, in contrast, uses a thermistor placed in the systemic arterial circulation, typically the femoral artery [56]. The indicator travels through the right heart, pulmonary vasculature, left heart, and aorta before reaching the thermistor. According to manufacturers, the temperature loss across the thoracic vasculature enables derivation of volumetric variables reflecting cardiopulmonary function, such as global end-diastolic volume (*GEDV*) and extravascular lung water (*EVLW*). This method estimates global CO – i.e., the total forward flow through both the pulmonary and systemic circuits. As such, it does not differentiate between right- and left-sided cardiac dysfunction, nor does it allow direct assessment of *PAP* or *SvO₂.*

Both methods require central venous access and allow for measurement of *ScvO*₂. However, only PAC-based monitoring enables direct measurement of *SvO₂*, *PAP*, and *PAWP*.

### Non-invasive methods

These methods include a wide array of ultrasound-based and biosensor-based techniques [57]. Echocardiography remains foundational and can be extended with adjuncts like lung ultrasound or venous congestion scores (e.g., VExUS) [58]. Other systems employ bioimpedance or bioreactance to infer hemodynamic variables [57]. Devices using plethysmographic signals from digital perfusion also exist but vary in accuracy and validation. Accuracy and precision, particularly in critically ill patients, remain unclear [6, 59].

### Advantages, limitations, and selection of monitoring modalities

The choice of hemodynamic monitoring device should be driven by the clinical question, not by technological availability. PAC provides comprehensive information including *PAP*, *PAWP*, *SvO₂* and, with contemporary catheters, continuous *CO* and right ventricular performance indices, but at the price of invasiveness and procedure-related risk [60]. Transpulmonary thermodilution systems offer global volumetric indices (*GEDV*, *EVLW*) and calibrated *CO* with lower invasiveness but do not distinguish right- from left-sided dysfunction and require central venous and arterial access. Uncalibrated pulse contour methods are less invasive and provide continuous trends of *CO* and dynamic preload indices, yet their accuracy depends on vascular tone and may deteriorate during vasoplegia or rapid changes in afterload. Echocardiography is non- or minimally invasive, provides direct structural information and qualitative assessment of systolic function, but is intermittent and operator-dependent. Bioimpedance and bioreactance are attractive non-invasive alternatives but show variable accuracy in critically ill patients. In the operating room, brief episodes of hemodynamic instability during intermediate-risk surgery may be adequately managed with intermittent echocardiography, dynamic preload indices, and uncalibrated pulse contour analysis. In high-risk surgery (e.g. major oncologic, cardiac, complex vascular procedures) or in patients with severe pre-existing cardiac disease, calibrated modalities such as transpulmonary thermodilution or, in selected cases, PAC monitoring can improve diagnostic precision and guide complex fluid and vasoactive strategies. In the ICU, persistent shock of unclear mechanism, severe right ventricular failure, pulmonary hypertension, or complex mixed shock states are typical indications for PAC-based monitoring, whereas transpulmonary thermodilution may be preferable when volumetric preload assessment and *EVLW* are central questions. In contrast, for short-lived, less severe instability, a combination of high-quality echocardiography and non-invasive or minimally invasive *CO* trends is often sufficient.

### Intraoperative hemodynamic monitoring strategies

The decision regarding the choice of intraoperative hemodynamic monitoring should consider three key domains: the anesthetic technique, the individual patient, and the surgical procedure (Table 4). Due to the dynamic nature of surgery and the constraints imposed by the procedure itself, not all monitoring modalities may be feasible in the intraoperative setting. Moreover, the invasiveness and economic cost of each method must be weighed against its expected clinical benefit. Because expanding the scope of monitoring during surgery is often neither simple nor rapid, decisions regarding perioperative hemodynamic management must be made proactively – ideally during preoperative planning. Volume management, as well as the interaction between surgical and anesthetic factors in patients with pre-existing cardiac disease, remains a central focus of intraoperative hemodynamic care. While liberal fluid strategies increase the risk of volume overload and tissue edema, overly restrictive regimens may compromise tissue perfusion. Despite the lack of a clear definition [23], which hampers comparability across studies and institutions, the overarching aim of GDT is to guide the clinical decision-making process regarding fluid resuscitation, inotropic support, and vasopressor therapy using hemodynamic variables. Although several randomized trials have evaluated GDT algorithms in specific surgical populations, the results have been mixed. One challenge in interpreting and generalizing these findings is the lack of standardization across monitoring devices: although different systems may refer to the same variables (e.g., stroke volume variation, *CI*), it remains uncertain to what extent these values are interchangeable across manufacturers [12–15]. In high-risk patients or during major surgical procedures (e.g., cardiac surgery), the use of advanced hemodynamic monitoring is associated with improved outcomes [61–64]. It enables timely therapeutic interventions and helps avoid both hypovolemia and fluid overload. Early studies by Shoemaker et al. [16] suggested that achieving supranormal oxygen delivery through invasive hemodynamic optimization could reduce postoperative complications, although these findings were later criticized for methodological limitations. The OPTIMISE trial evaluated the use of *CO*-guided fluid and inotrope therapy in high-risk surgical patients but did not show a significant reduction in the composite outcome of complications and mortality [65]. OPTIMISE II focused on the use of low-dose dobutamine in addition to fluid therapy in major gastrointestinal surgery but again failed to demonstrate a clear survival benefit [19]. In contrast, the intervention group exhibited a higher rate of acute cardiac events within 24 h: 3.0% in the intervention group vs. 1.7% in the control group (OR 1.82; *p* = 0.03). Similarly, the iPEGASUS trial assessed effect of maintaining optimized postinduction *CI* in major abdominal surgery [18]. The 28-day composite primary outcome occurred in 55% in the *CI*-guided group versus 46% in the routine care (adjusted OR 1.84; *p* = 0.047). Taken together, these studies suggest that while perioperative GDT is physiologically sound and may benefit selected high-risk patients, its broad application does not consistently improve hard clinical outcomes. The implementation of GDT should therefore be individualized and integrated into a broader strategy of perioperative risk management.

**Table 4:** Domains informing the choice of intraoperative hemodynamic monitoring

Domain	Consideration
Anesthetic Technique	
	Anesthesia induced Vasodilatation
	Positive Presssure Ventilation
	One-Lung Ventilation
Patient Factors	
	Cardiopulmonary Comorbidity
	Right Heart vs. Left Heart Comorbidity
	Preload Dependency/Volume Status
	Functional Reserve (e.g. Frailty)
Surgical Procedure	
	Endoscopic vs. Conventional Surgery
	Anticipated blood/fluid loss
	Positioning Effects (e.g. Trendelenburg, Beach-Chair)
	Need for real-time volume or pressure management (e.g. neurosurgical surgery)
	Threat of air or fat embolism or palacos reaction

This table highlights three major domains that should be considered when selecting the appropriate intraoperative hemodynamic monitoring strategy: the anesthetic technique, the individual patient, and the surgical procedure. Each domain includes specific examples and considerations that may influence monitoring needs. These include anesthesia-induced vasodilation, cardiopulmonary comorbidities and volume responsiveness, as well as surgical factors such as blood loss, positioning, or the risk of embolic events. A tailored monitoring approach based on these interacting factors helps ensure adequate perfusion and patient safety

### Hemodynamic monitoring in the intensive care unit

In the ICU, the spectrum of hemodynamic instability is broader, more severe, and often sustained over longer periods of time. As a result, both therapeutic strategies and the monitoring of disease trajectories must be adapted accordingly. Conditions such as septic shock, cardiogenic shock, and acute respiratory failure require individualized, pathophysiology-based management. A central aspect of ICU hemodynamic care is the ongoing assessment of whether *DO₂* adequately meets metabolic demand, as well as the identification of the underlying cause in cases of mismatch – whether cardiac, hypovolemic, or vasoplegic in origin. Septic shock is typically characterized by peripheral vasodilation and relative hypovolemia. Initial management requires prompt fluid resuscitation, followed by vasopressor therapy – most commonly norepinephrine – to restore *MAP* ≥ 65 mmHg. Continuous assessment of fluid responsiveness is crucial to avoid unnecessary volume overload, which is now a key principle in sepsis management. In contrast, cardiogenic shock necessitates inotropic support to augment myocardial contractility and, in selected cases, mechanical circulatory assistance. Hemodynamic targets in the ICU must be dynamic and responsive to clinical evolution. Rather than aiming for rigid absolute values, trends and responses to interventions provide more meaningful guidance. For instance, rising lactate levels may signal deteriorating perfusion and prompt escalation of therapy, whereas an improving *ScvO₂* suggests restored circulatory adequacy.

When feasible, continuous monitoring is preferable to intermittent measurement, particularly in unstable patients. The use of structured reassessment protocols – such as evaluating fluid responsiveness every four hours – can improve consistency and quality of care. In the intensive care unit, the GDT concept has been extensively studied across various clinical contexts. In sepsis, the landmark Rivers et al. trial [66] introduced early goal-directed therapy (EGDT), showing a significant mortality reduction from 46% to 30%. Multicenter trials – ProCESS [67], ARISE [68], and ProMISe [69] – failed to replicate these findings in modern ICU settings with timely standard care, casting doubt on the universal benefit of protocolized EGDT. These trials suggest that early identification and treatment remain critical, but rigid protocols offer limited additional advantage when standard care is already prompt and adequate. Several factors likely explain the discrepancy between the original Rivers EGDT trial [66] and the subsequent ProCESS [67], ARISE [68], and ProMISe [69] studies. First, baseline *ScvO*₂ at randomization differed markedly. In Rivers’ trial [66], mean *ScvO₂* at inclusion was ~ 49%, indicating profound supply-demand mismatch and a large potential for benefit from *DO₂* optimization. In contrast, in ProCESS [67], ARISE [68], and ProMISe [69] the first recorded *ScvO₂* values were already around 70–72%, well within or close to the target range, reflecting substantial resuscitation before randomization and limiting the incremental value of protocolized *ScvO₂*-driven interventions [70]. Second, usual care in the more recent trials incorporated early antibiotics, timely fluid resuscitation, and vasopressor use, thereby narrowing the gap between protocolized and non-protocolized management [70]. Third, differences in transfusion thresholds and reliance on CVP as a fluid target further question the external validity of the original EGDT protocol in contemporary practice. These considerations support the notion that rigid EGDT algorithms offer little additional benefit when high-quality standard care is already in place, and they reinforce the need for individualized, physiology-guided resuscitation rather than protocol adherence per se. In patients with aneurysmal subarachnoid hemorrhage, GDT has been proposed to prevent delayed cerebral ischemia by optimizing volume status and cerebral perfusion pressure. In a prospective randomized controlled trial involving 108 patients GDT reduced occurrence of delayed cerebral ischemia to 13% compared with 32% in the control group (adjusted HR 2.84; *p* = 0.02) [71]. Still, robust evidence for improved neurological outcomes remains lacking. Current practice favors pragmatic, physiology-driven interventions tailored to the phase of SAH and the risk of vasospasm, rather than protocolized GDT [72]. In the context of solid organ transplantation, particularly liver transplantation, GDT aims to mitigate profound hemodynamic shifts and maintain end-organ perfusion. A secondary outcome analyses of the COLT trial investigated individualized hemodynamic therapy after liver transplantation using continuous *CO* monitoring and vasopressor/inotrope titration [73]. While it demonstrated feasibility and hemodynamic stability, there was no significant difference in major postoperative complications. Nevertheless, smaller studies suggest that GDT may reduce intraoperative fluid overload and transfusion requirements, supporting its selective use in high-risk procedures.

Collectively, these findings indicate that while the physiologic rationale for goal-directed therapy remains compelling, its impact on clinical outcomes in the ICU is highly context-dependent. GDT should not be viewed as a universal solution but rather as one component of individualized, physiology-guided critical care.

## Conclusion

Effective hemodynamic management depends on the integration of clinical assessment, physiological principles, and the appropriate application of monitoring technologies. Static variables are increasingly being replaced by dynamic, patient-specific assessments that more accurately reflect the circulatory status.

In both surgical and intensive care settings, individualized and responsive hemodynamic strategies have been shown to improve patient outcomes and reduce complications. As monitoring technologies continue to evolve, it is ultimately the clinician’s understanding of cardiovascular physiology that determines the successful translation of data into effective therapy.

Ongoing education, hands-on ultrasound training, and a structured approach to hemodynamic evaluation are essential pillars of modern perioperative and critical care medicine.

## Data Availability

No datasets were generated or analysed during the current study.

## Abbreviations

avDCO₂: Arterial–venous carbon dioxide difference
CI: Cardiac index
CO: Cardiac output
CVC: Central venous catheter
DO₂: Oxygen delivery
ECMO: Extracorporeal membrane oxygenation
EGDT: Early goal-directed therapy
ELWI: Extravascular lung water index
EVLW: Extravascular lung water
GEDV: Global end-diastolic volume
GEDVI: Global end-diastolic volume index
GDT: Goal-directed therapy
IABP: Intra-aortic balloon pump
ICU: Intensive care unit
LV: Left ventricle
MAP: Mean arterial pressure
MCFP: Mean circulatory filling pressure
MPP: Mean Perfusion Pressure
MSFP: Mean systemic filling pressure
PAC: Pulmonary artery catheter
PAWP: Pulmonary artery wedge pressure
PAP: Pulmonary artery pressure
PVR: Pulmonary vascular resistance
RAP: Right atrial pressure
RRT: Renal replacement therapy
REF: Reference (used as placeholder for missing citation)
R_v_: Resistance to venous return
SAH: Subarachnoid hemorrhage
ScvO₂: Central venous oxygen saturation
SV: Stroke volume
SVR: Systemic vascular resistance
SVRI: Systemic vascular resistance index
SvO₂: Mixed venous oxygen saturation
V_eff: Effective circulating volume
VO₂: Oxygen consumption
VR: Venous return

## Declarations

### AI statement

During the preparation of this manuscript, the authors employed ChatGPT (OpenAI, San Francisco, CA, USA) to assist in refining language, grammar, and style. All content generated by the tool was thoroughly reviewed, verified, and edited by the authors, who take full responsibility for the accuracy and integrity of the final manuscript.
